# Metal-Free Photocatalysis: Two-Dimensional Nanomaterial Connection toward Advanced Organic Synthesis

**DOI:** 10.1021/acsnano.1c00627

**Published:** 2021-03-14

**Authors:** Cristian Rosso, Giacomo Filippini, Alejandro Criado, Michele Melchionna, Paolo Fornasiero, Maurizio Prato

**Affiliations:** † Department of Chemical and Pharmaceutical Sciences, CENMAT, Center of Excellence for Nanostructured Materials, INSTM, UdR Trieste, University of Trieste, Via Licio Giorgieri 1, Trieste 34127, Italy; ‡ Center for Cooperative Research in Biomaterials (CIC biomaGUNE), Basque Research and Technology Alliance (BRTA), Paseo de Miramón 182, 20014 Donostia San Sebastián, Spain; § ICCOM-CNR Trieste Research Unit, University of Trieste, Trieste 34127, Italy; ∥ Basque Foundation for Science, Ikerbasque, Bilbao 48013, Spain

## Abstract

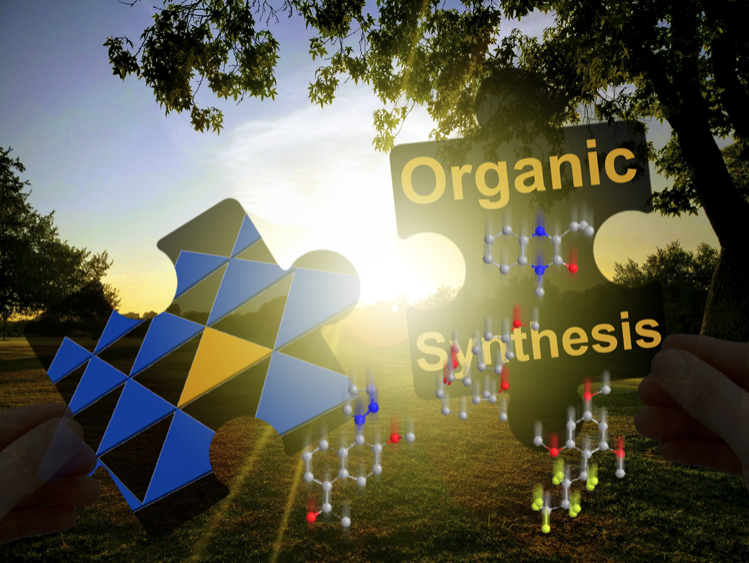

Two-dimensional (2D) nanostructures are a frontier in materials chemistry as a result of their extraordinary properties. Metal-free 2D nanomaterials possess extra appeal due to their improved cost-effectiveness and lower toxicity with respect to many inorganic structures. The outstanding electronic characteristics of some metal-free 2D semiconductors have projected them into the world of organic synthesis, where they can function as high-performance photocatalysts to drive the sustainable synthesis of high-value organic molecules. Recent reports on this topic have inspired a stream of research and opened up a theme that we believe will become one of the most dominant trends in the forthcoming years.

Although research on two-dimensional (2D) materials traces back several decades, its renaissance can be pinpointed to when single-layer graphene was isolated and characterized by Geim and Novoselov, who were awarded the Nobel Prize in Physics for their discoveries.^1,2^ Since then, graphene’s incredible properties have inspired many researchers investigating a wide range of possible applications. The recognition that such distinct properties go hand in hand with the 2D arrangement motivated and accelerated the exploration of other 2D materials, which include both metal-based and metal-free materials. In recent years, research on 2D metal-free materials has become increasingly broad because of the lower materials cost of metal-free materials, as compared to that of metal-based structures. Among the various applications for such materials, photocatalysis is an exceptionally attractive field, fitting most of the aspects of the “green chemistry” modern philosophy, where integrating sustainability criteria into chemical production is the core mission. In this context, exploiting the energy of solar light to trigger chemical transformations in lieu of more energy-intensive and less ecological production schemes represents a big step forward toward sustainability.^3,4^ Despite the promising findings and the high expectations for the use of 2D metal-free materials as photocatalysts for organic transformations, the full potential of these intriguing structures has yet to be uncovered, and understanding the structure/activity relationship still requires a great deal of investigation. In this Perspective, we identify the critical points of 2D metal-free materials and discuss their success as photocatalysts for advanced organic synthesis. We also offer critical discussion on the areas to be improved to extend applicability and increase industrial appeal. Finally, we present emerging trends in 2D materials photocatalysis leading toward richer organic synthesis.

## Types of Two-Dimensional Materials

Although different classes of 2D materials exist, 2D materials are often described as layered solids with a high in-plane bond strength but weak interplanar interactions, typically deriving from van der Waals forces.^5^ The layered structure can be exfoliated into thinner, few-layer structures with relative ease.^6−8^ A more stringent definition of 2D materials is restricted to those featuring single-atom-thick layers, whereby the resemblance with relativistic Dirac particles makes them unique.^9^ In contrast with monolayered 2D species, few-layered 2D materials are more accessible and versatile, explaining their popularity from an application-focused point of view. The flexibility in defining 2D materials has led to a “gray area”, where the arrangement of chemical species organized in sheets has been taken as an indicator to claim the 2D nature, even when multilayered structures are the subject of the reported study. It is important to keep in mind that the two situations (thin, few-layered *vs* multilayered bulk solids) usually generate dramatic changes in the material properties.

## Why Focus on Two-Dimensional Structures for Photocatalysis?

**Figure 1:**
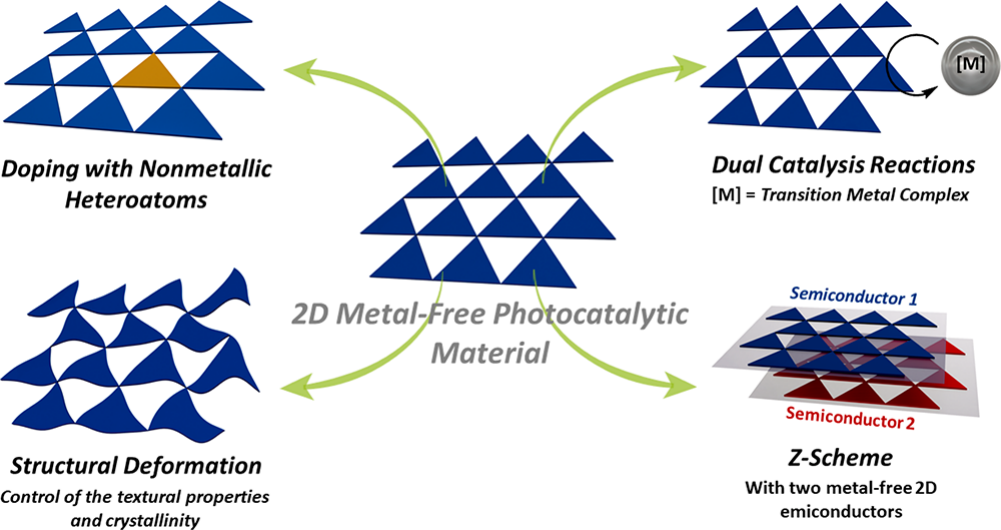
Graphical representation of the foreseen major avenues to organic photocatalysis by metal-free two-dimensional (2D) materials.

The properties of 2D photocatalysts match some photocatalysis requirements well. The planar sizes in 2D materials can reach the micron scale, with concomitant enhancement of the specific surface area.^10^ In addition, thicknesses can be reduced to a few nanometers, or even, in some cases, to monatomic sizes, using modern synthetic approaches.^11^ These geometric features are notably correlated with quantum and dielectric confinement effects, which modify the band structure and, consequently, the band gap.^12^ Typically, the band gap widens due to quantum confinement, and upshifts of the conduction band (CB) are observed, enhancing both the potential energy of the photogenerated electrons and their reduction ability.^13^ The 2D geometry also improves the separation and migration of the charge carriers, which are prerequisites for efficient photocatalysis, and higher densities of surface active sites. Moreover, the chemical and morphological structures can be locally modified, enabling tunability of the defect density.^14,15^ It is also possible to adjust the electronic states by doping the lattice with various elements, either metals or nonmetals. Although doping is a versatile approach that has also been adopted for bulk catalytic materials, this strategy notably benefits from the 2D arrangement for reaching (i) higher per-mass relative dopant concentrations and (ii) superior control over the dopant environment. The former aspect capitalizes on the easier diffusion of the dopant through a thinner structure (as compared to bulk materials), whereas the latter benefits from the usually higher homogeneity of 2D layered materials.^15^ Finally, great opportunities arise from creating interfaced 2D structures by combining two different phases. This combination results in the creation of heterojunctions (p–n) or *Z*-schemes, which are two of the most modern approaches for achieving significant enhancement of catalytic performance.^16^ Metal-free interfaces are also a rapidly emerging field, with 2D hybrid structures offering various advantages.^17^ It is evident that the effectiveness of the interface is maximized in 2D structures for geometric reasons, which also enables the construction of devices with higher mechanical flexibilities (Figure 1).^18^

Although metal-free 2D materials can be based on several elements including Si, Se, P, S, B, or Te, carbon has been investigated the most for the design and synthesis of photocatalysts. Carbon has been preferred to date because of its easy availability and the rich arsenal of available morphologies of carbon materials, particularly at the nanoscale, which enables tailoring of the physicochemical and electronic properties.^19^ For instance, these properties can be tuned by simple chemical modification of graphene *via* introduction of functional groups or dopants.^20−25^ Apart from carbon, other nonmetal 2D materials with semiconducting properties that have emerged include hexagonal boron nitride (h-BN) and black phosphorus (BP), although their use has thus far mainly involved energy-related catalysis.^26−28^ A key issue when reporting metal-free catalysts is to ascertain that no adventitious metal impurities are incorporated within the material, as even at low parts per million (ppm) levels metals can affect the performance, thus generating false conclusions and reproducibility problems.^29^

## Two-Dimensional Metal-Free Photocatalysts: Protagonists, Minor Characters, and Rising Stars

**Figure 2:**
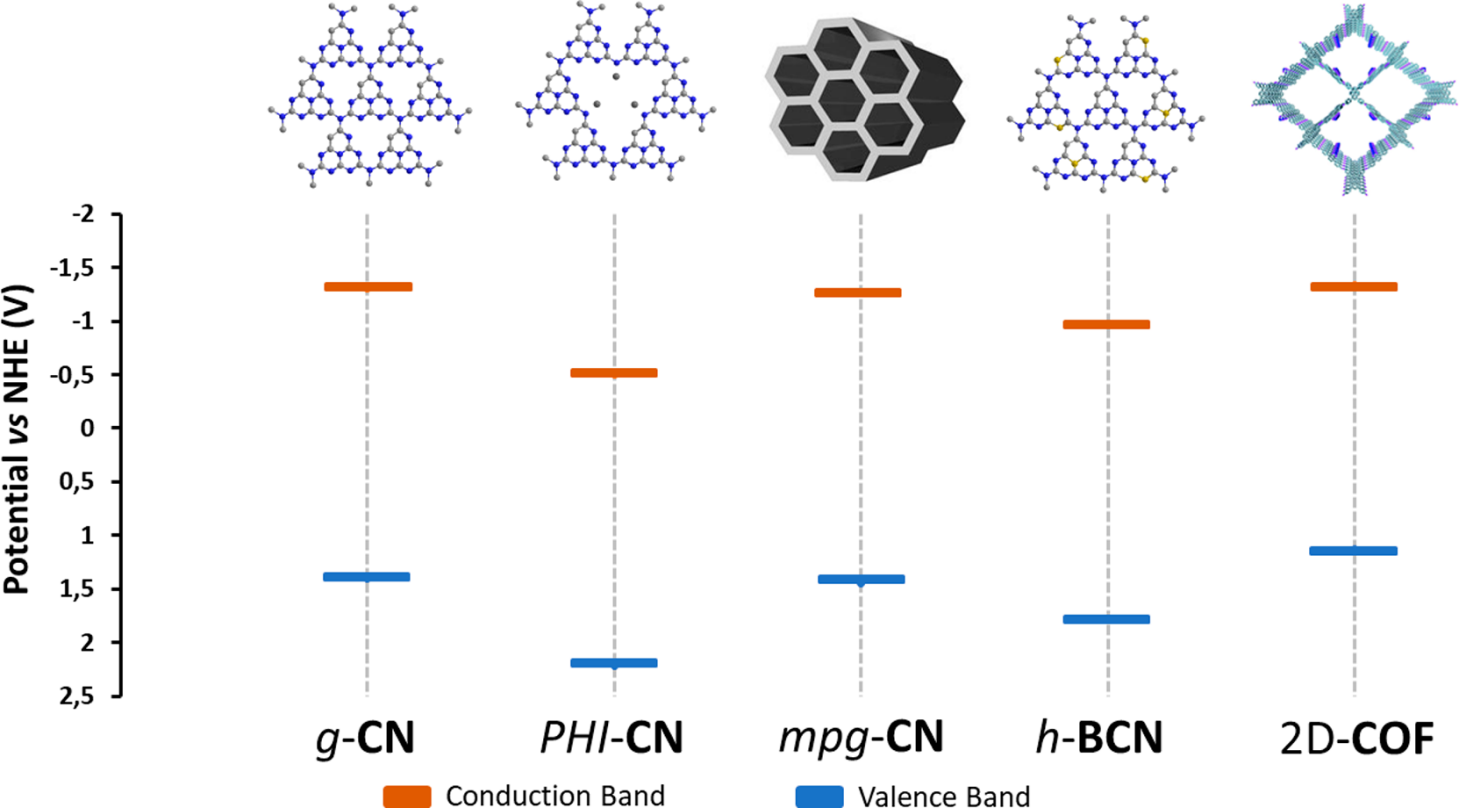
Typical band edge potentials of the two-dimensional metal-free semiconductors graphitic-CN (g-CN), poly(heptazine imides) (PHI-CN), mesoporous carbon nitride (mpg-CN), hexagonal boron carbon nitride (h-BCN), and two-dimensional covalent organic frameworks (2D-COFs) discussed herein.

Among 2D structures (Figure 2), the carbon nitrides (CNs) represent the most popular choice for photocatalytic applications, including organic transformations, because of their visible-light absorption, facile synthesis, stability, and versatility for structural modifications.^30−32^

**Figure 3:**
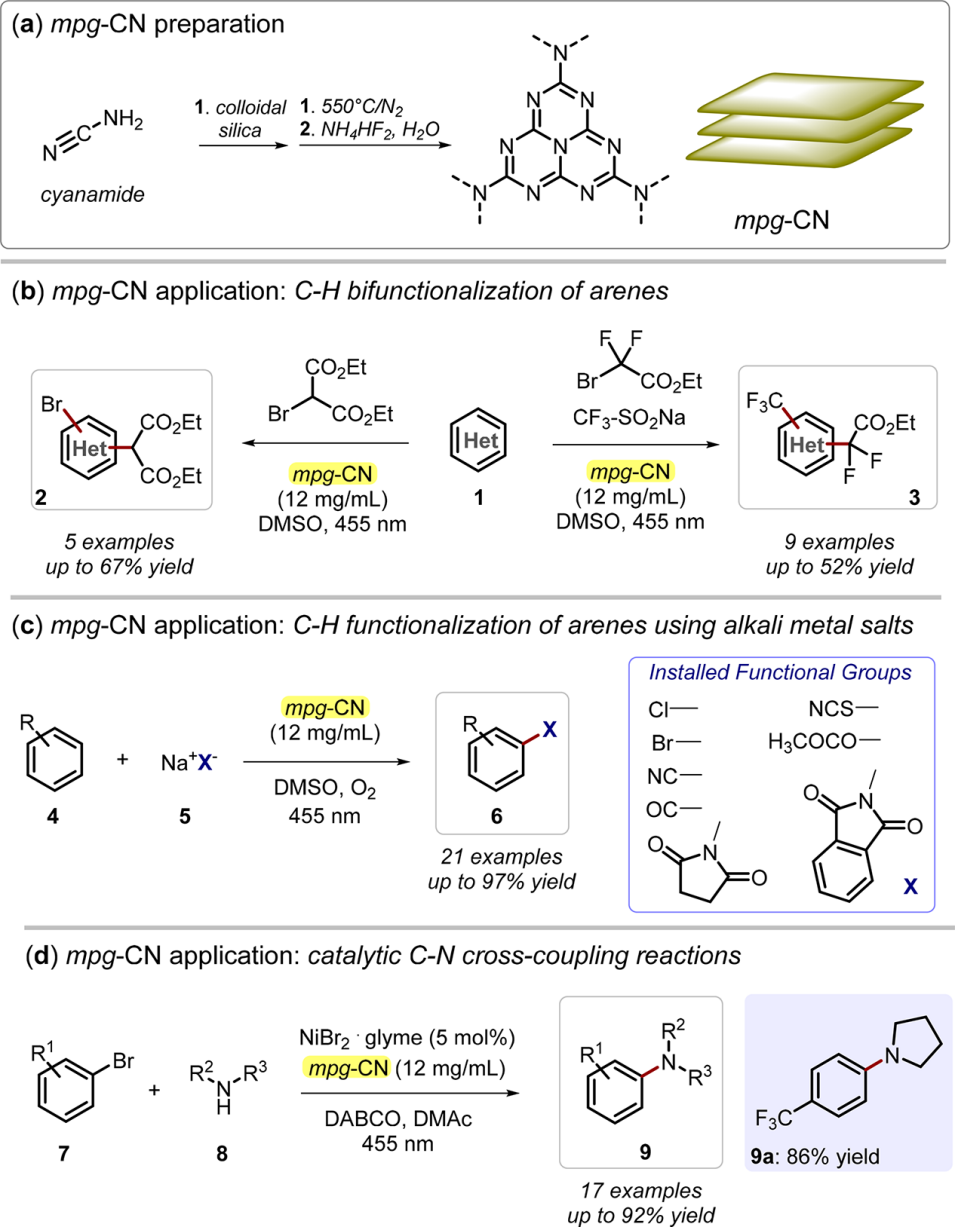
(a) Mesoporous carbon nitride (mpg-CN**)** preparation from cyanamide through thermal treatment. (b) mpg-CN application in C–H bifunctionalization of arenes and heteroarenes. (c) mpg-CN application in C–H functionalization of arenes using alkali metal salts. (d) mpg-CN application in C–N cross-coupling reactions. DMSO: dimethyl sulfoxide.

Carbon-nitride-based materials diversify to large extents, and various structures have been reported, building a portfolio of material subclasses. From a structural point of view, the most stable and most frequently investigated allotrope, namely, graphitic-CN (g-CN), is proposed to be constituted by repeating N-bridged poly(tri-*s*-triazine) frameworks arranged into graphite-like π-conjugated planar layers (although other repeating units have been proposed, such as, for example, *s*-triazine).^33,34^ The C/N ratio in g-CN is theoretically 3/4 (as indicated by the typically used formula, C_3_N_4_); however, the experimental ratio deviates from this value depending on the synthetic procedure due to the formation of defective structures and incorporation of other elements (*e.g.*, O). The accurate structure of g-CN (and other CNs in general) has not been defined in detail, and often the depiction of the structure is merely a simplification for guiding the reader. Because conventional preparation protocols (which are also the simplest ones) based on pyrolysis of solid precursors do not enable easy control on the final structure, detailed knowledge of the entire structure is not accessible in most cases. This lack of knowledge poses an extra challenge for theoretical studies on CN catalysts, as the observed activity may rely on definite structural features (defects, specific moieties, or others) that could be overlooked during computational analyses, thus generating erroneous conclusions. To mitigate this problem, diligent and in-depth characterization of the materials should become a routine part of the work, even when materials syntheses are replicated from previously published articles, because marginal differences in conditions may lead to alterations of the final structures. Fortunately, rapid progress is being made, leading to next generations of materials with improved performance and better-defined structures.^35^ The position of both valence and conduction bands (VB and CB, respectively) can be modulated on the basis of the C/N ratio, polymerization degree, crystallinity, and the presence of doping agents (*e.g.*, boron, sulfur, phosphorus, organic additives).^30,34,36^ The textural properties can also be tailored for better photocatalytic performance, as shown by the development of mesoporous carbon nitride (mpg-CN), which possesses surface areas much higher than those of g-CN.^37,38^ Indeed, mpg-CN has recently emerged in organic reactions for the synthetically relevant functionalization of arenes and heteroarenes (Figure 3).^39^

**Figure 4:**
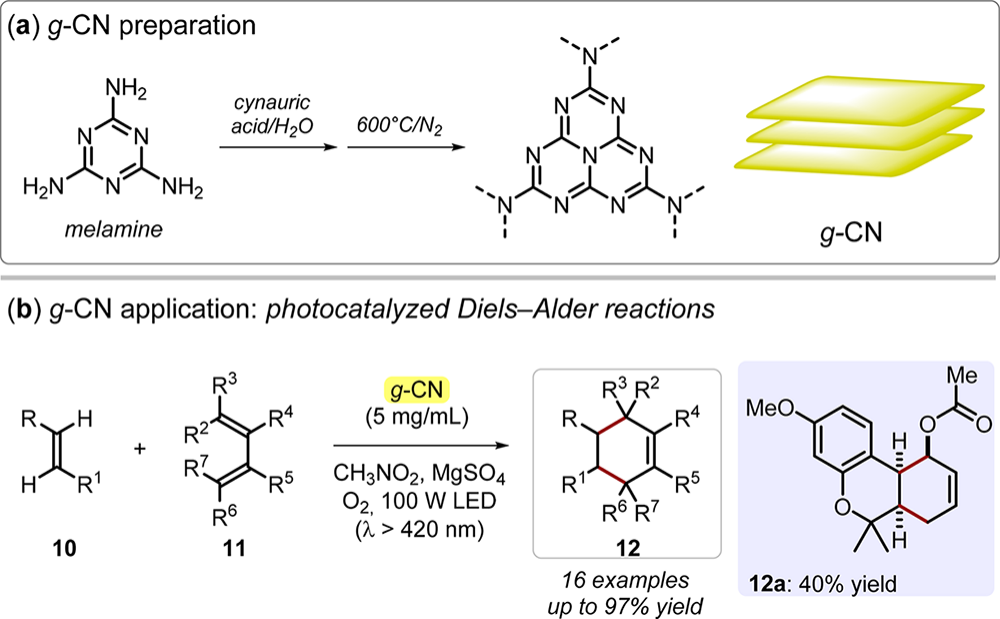
(a) Graphitic carbon nitride (g-CN) preparation from melamine and cyanuric acid through pyrolysis. (b) g-CN application in photocatalyzed Diels–Alder reactions. LED: light-emitting diode.

Although CN materials are conventionally prepared as bulk, appropriate protocols for 2D structures have been proposed. For example, Zhao and Antonietti showed that, starting from melamine and cyanuric acid, a g*-*CN consisting of thin, multisheet structures (thicknesses in the range of 15–20 nm) could be prepared. This catalyst effectively promoted a photocatalyzed Diels–Alder reactions under visible light irradiation,^40^ being one of the milestones in 2D CN-based photocatalytic organic synthesis (Figure 4).

**Figure 5:**
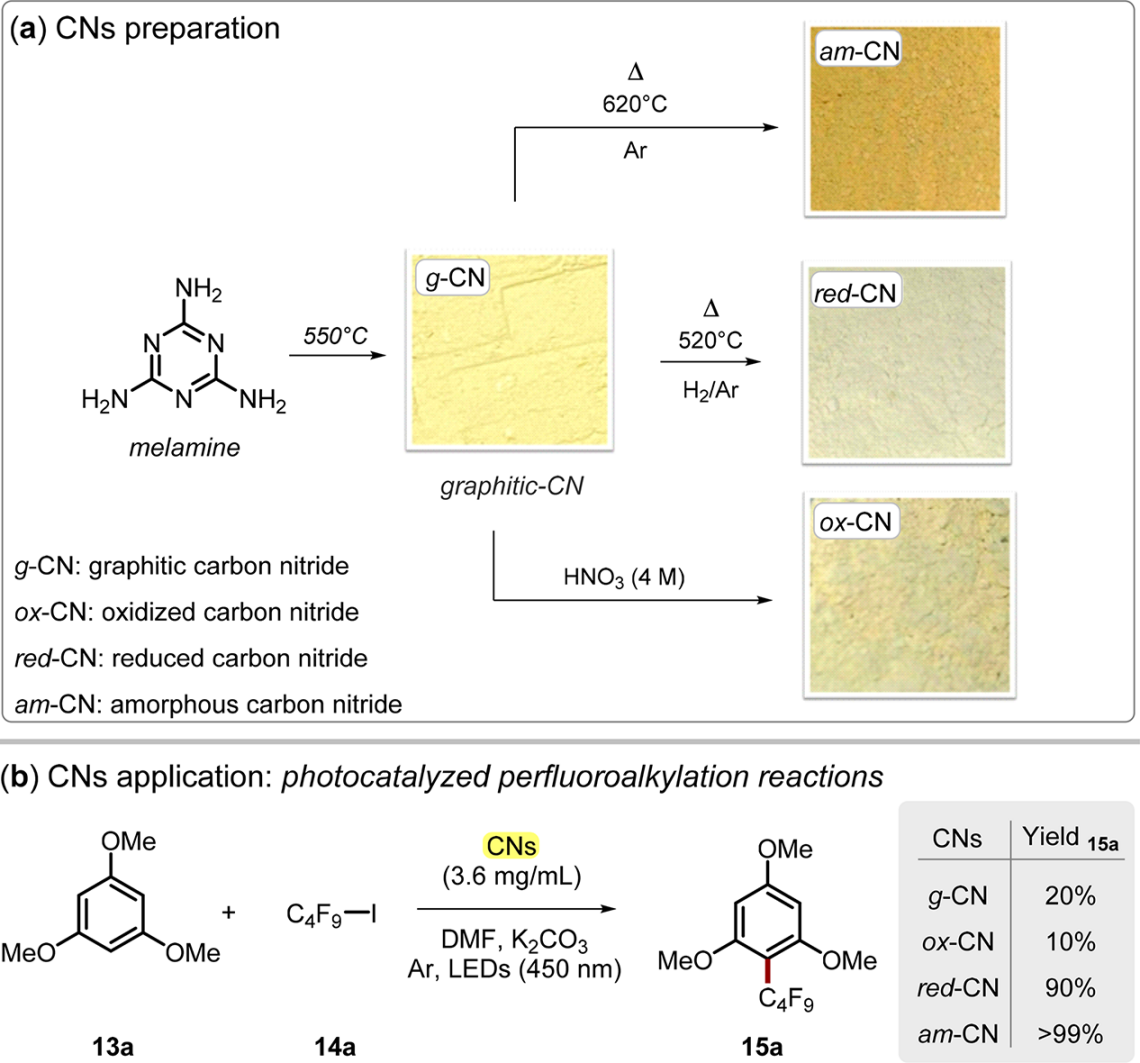
(a) Carbon nitride (CN) preparation from melamine and related postsynthetic modifications. (b) CN applications in photocatalyzed perfluoroalkylation reactions.

Understanding the textural properties of this material could have provided additional information related to activity, and we propose that future studies should carefully consider the contributions of surface area and pore size distributions.^41^ A similar g-CN prepared by pyrolysis of guanidine was employed to carry out photo-oxidative additions of aminoalkyl radical precursors to unsaturated acceptors.^42^ Researchers have demonstrated that exfoliation of CN bulk materials into thin 2D nanosheets is one main contributing factors to the much enhanced photocatalytic activity, as a result of the increased active site density.^43,44^ Graphitic CN in its pristine form generally exhibits moderate catalytic activity because of the sluggish conductivity and low surface area. A great deal of research has focused on strategies to modify the pristine material in order to boost the resulting photocatalytic activity.^35^ In this direction, our group recently investigated how postsynthetic modifications of g-CN can influence the outcome of a photocatalytic process, namely, perfluoroalkylation reactions of electron-rich organic molecules (Figure 5).^45^

Important insights into the mechanistic features were gathered by advanced ^19^F nuclear magnetic resonance techniques, revealing the criticality of the fluorinated substrate’s affinity toward the CN surface. In our opinion, studies that couple the screening of new organic reactions with investigations (theoretical and experimental) on the related mechanism could considerably accelerate the development of 2D photocatalysts toward industrial feasibility.

One subclass of CN that is flourishing in photocatalytic applications are poly(heptazine imides) (PHI-CN). These materials are conventionally prepared by eutectic molten salts methods, giving rise to a nanometer-size layered structure with continuous channels. The synthesis generates some negatively charged N sites, which bind the cation of the employed metal salt (usually K^+^ or Na^+^).^46^ Although most of the CN photocatalysts have always been linked to electron-transfer processes, PHI-CN is also capable of driving energy-transfer reactions. This ability can translate into the generation of excited-state molecules other than charged radicals, paving the way for new reactivities.^47−51^ Moreover, PHI-CN materials offer another interesting opportunity in that the alkaline metals could be replaced by other transition metals through cation exchange strategies, thus potentially leading to CN–single atom materials.^52^

**Figure 6:**
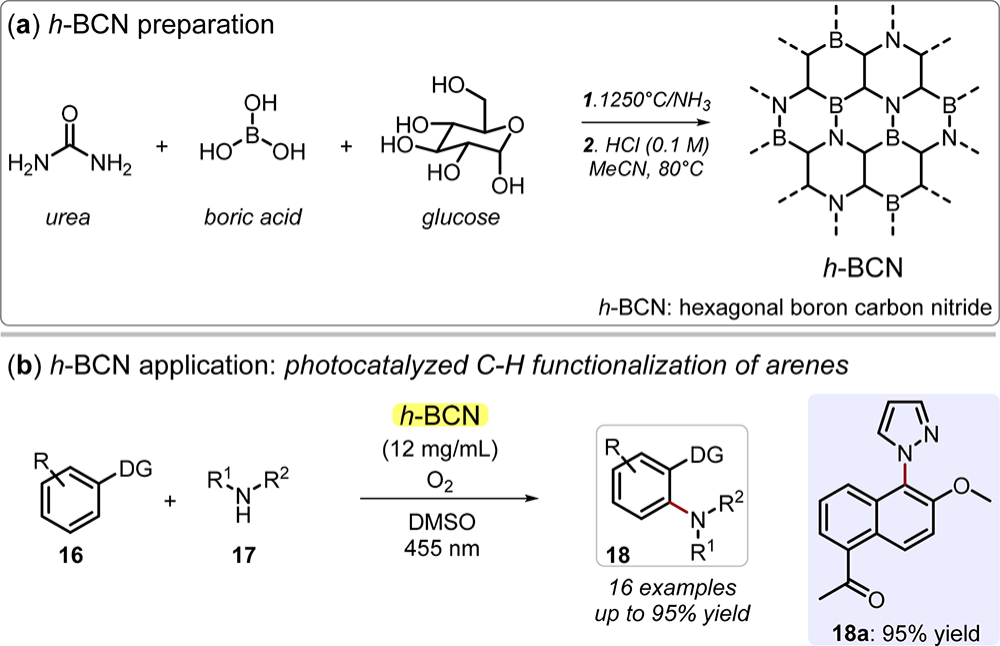
(a) Hexagonal boron carbon nitride (h-BCN) preparation from urea, boric acid, and glucose through calcination. (b) h-BCN application in photocatalyzed C–H functionalization of arenes. DG: directing group.

Although they have been less investigated to date, boron carbon nitrides (BCNs) also offer interesting potential applications. Boron CNs are ternary-component materials made of carbon, nitrogen, and boron that can be formed with cubic (c-BCN) or hexagonal (h-BCN) crystal structures. In particular, h-BCN synthesis resulted from interest in combining graphene with hexagonal boron nitride (h-BN) to amend the 0 band gap of graphene (G) and the wide band gap of h-BN (typically above 5 eV) simultaneously. As theoretical studies have confirmed,^53^ the resulting material (h-BCN) shows an intermediate optical behavior with absorption energy that can be adjusted in the visible range (*e.g*., by varying the BCN stoichiometry),^54^ with the possibility of forming segregated domains of one of the three elements (typically C).^55,56^ As a result, h-BCN is an appealing option for photocatalytic applications,^57^ and, apart from water splitting evolution and CO_2_ reduction,^56,57^ h-BCN has recently attracted attention as a catalyst for synthetically relevant photoredox reactions.^58,59^ König, Wang, and co-workers studied the photochemical C–H functionalization of electron-rich arenes catalyzed by an h-BCN with notable activity,^60^ whereas other photo-oxidation and photoreduction reactions were possible by simply tuning the relative content of the h-BCN precursors (typically glucose and boric acid, Figure 6).^61−63^ Despite the above-mentioned encouraging case studies, organic photocatalysis by BCN is still in its infancy; therefore, there is fertile soil for future developments.

**Figure 7:**
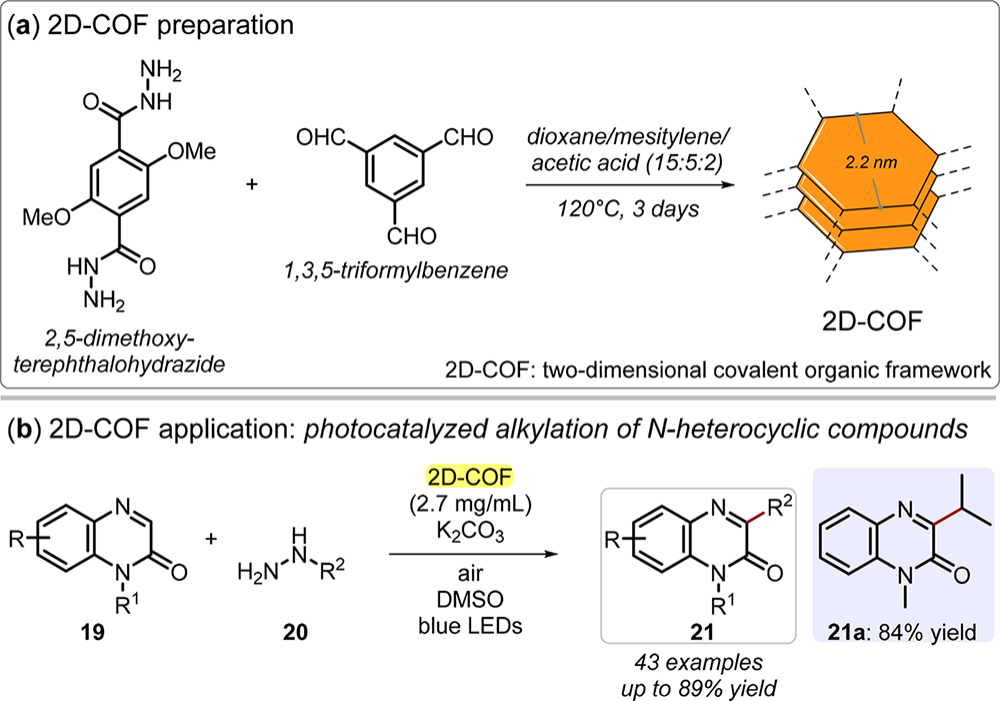
(a) Two-dimensional covalent-organic framework (2D-COF) preparation from 2,5-dimethoxyterephthalohydrazide and 1,3,5-triformylbenzene through solvothermal treatment. (b) 2D-COF application in photocatalyzed alkylation of N-heterocyclic compounds.

Finally, covalent organic frameworks (COFs) are a popular class of single-phase 2D metal-free candidates for photocatalytic organic synthesis. Covalent organic frameworks are covalent, porous crystalline polymers that enable the integration of organic motifs into an ordered structure.^64,65^ The better-defined structures of COFs, as compared to those of CN or BCN, make this class of materials distinctive. In particular, 2D-COFs possess extended π-conjugated frameworks and eclipsed stacked sheets with regularly aligned columns, where the ordered columns in 2D-COFs provide ideal channels for charge carrier transport in the stacking direction.^66^ In addition, despite their heterogeneity, the controlled, high porosity of 2D-COFs ensures great accessibility to active sites, offering excellent catalytic performance and the potential for higher reaction selectivity by means of pore size tailoring.^65,67^ Thus, 2D-COFs could lead to the development of photoactive materials for optoelectronics, photovoltaics, and visible light photocatalysis.^67−70^ For COFs, the 2D *versus* 3D distinction is easier to define because it evolves from the simplified symmetry of the specific building blocks used to construct the framework.^64^ In general, 2D-COFs exhibit a richer topology than other 2D materials (*i.e*., hexagonal or tetragonal geometries of different sizes), leading to increasing interest in the development of synthetic strategies for the next generation of materials.^71^ For catalytic applications, there are additional opportunities for introducing organic groups within specific channel positions, endowing COFs with enhanced functionality.^72^ Wang and co-workers illustrated the photochemical oxidation of boronic acids using three different 2D-COFs having different shapes and channel dimensions (hexagonal or rhombic repetitive units with dimensions of 1.4–2.8 nm).^73^ Recently, Yang and co-workers envisaged the use of a hydrazone-based 2D-COF with a hexagonal pore system with dimensions of 2.2 nm for carrying out photochemical alkylation of N-heterocyclic compounds (Figure 7).^74^ In addition to these examples, many other photocatalytic procedures have been reported, some of which deal with photo-oxidation reactions of simple organic substrates such as alcohols, amines, and sulfides.^75−77^ Nevertheless, more complex reactions such as photodehalogenations, cross-couplings, and cyclizations have demonstrated and proven the great versatility of 2D-COFs.^78−82^ Therefore, we expect rapid proliferation of more challenging organic transformations by 2D-COF photocatalysts.

## Emerging Directions and Vision for the Future

**Figure 8:**
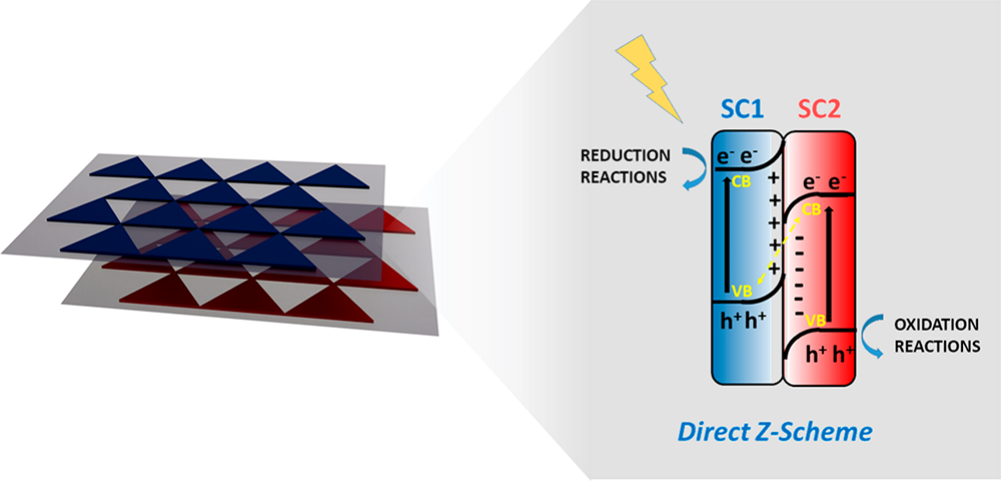
Graphical sketch of a *Z*-scheme and its function. Two semiconductors (SCs) opportunely interfaced and with staggered band configuration (and suitable band Fermi levels and work functions) give rise to band bending. The built-in internal electric field and the band bending cause coupling of the photogenerated holes and electrons of SC1 and SC2, respectively, whereas the electrons and holes in SC1 and SC2 are maintained and spatially separated, able to function for reduction and oxidation reactions.

Despite the great progress in photocatalytic organic synthesis by metal-free 2D single-phase catalysts, the parallelism with 2D inorganic hybrids, which, through creation of suitable heterojunctions, can remarkably enhance photocatalytic performance, has naturally provided a new direction for metal-free analogues. Specifically, one frontier is combining two metal-free phases and suitably interfacing them, thus preparing 2D metal-free nanohybrids to combine their catalytic behavior while exploiting the resulting new features. One objective is to replicate the well-known inorganic *Z*-schemes by relying on only nonmetal 2D structures. This method retards electron–hole recombination rates while exploiting the higher CB energy level and the lower VB energy level to enable coupled energetically demanding redox processes (Figure 8).^83^

The synergism in 2D metal-free nanohybrids for photocatalysis has thus far been confined to energy-related processes. A notable achievement was reported by He *et al.*, who prepared a 2D CN/h-BN nanohybrid by the direct growth of CN on h-BN nanosheets, which they successfully used for photocatalytic H_2_ and H_2_O_2_ synthesis. Enhanced activity originated from the suitable interfacial domain between CN and h-BN, causing physical separation of the charge carriers and prolonging their lifetime, although, in this case, the potential energy of the photoexcited electrons was reduced following the CN to h-BN injection.^84^ Two-dimensional BP/CN catalytic heterostructure for H_2_ evolution is another remarkable example of interfacial synergism, whereby charge transfer inhibited charge recombination, making catalysis possible, even under infrared irradiation.^85^ Other interesting examples with graphene/CN and BP**/**covalent triazine frameworks used for water splitting or decontamination of organic pollutants indicate the great potential of this type of heterostructure.^17,86,87^ We anticipate that their use in photocatalytic organic synthesis will soon take off, making the synthesis of high-value organic compounds with good solar-to-chemical efficiency possible. A thorough evaluation of the band structures and Fermi levels of the two phases will be critical to establish truly cooperative mechanisms, possibly by means of the *Z*-scheme configuration, and synthetic efforts must look at the phase connection, maximizing interfacial domains with strong interactions.

**Figure 9:**
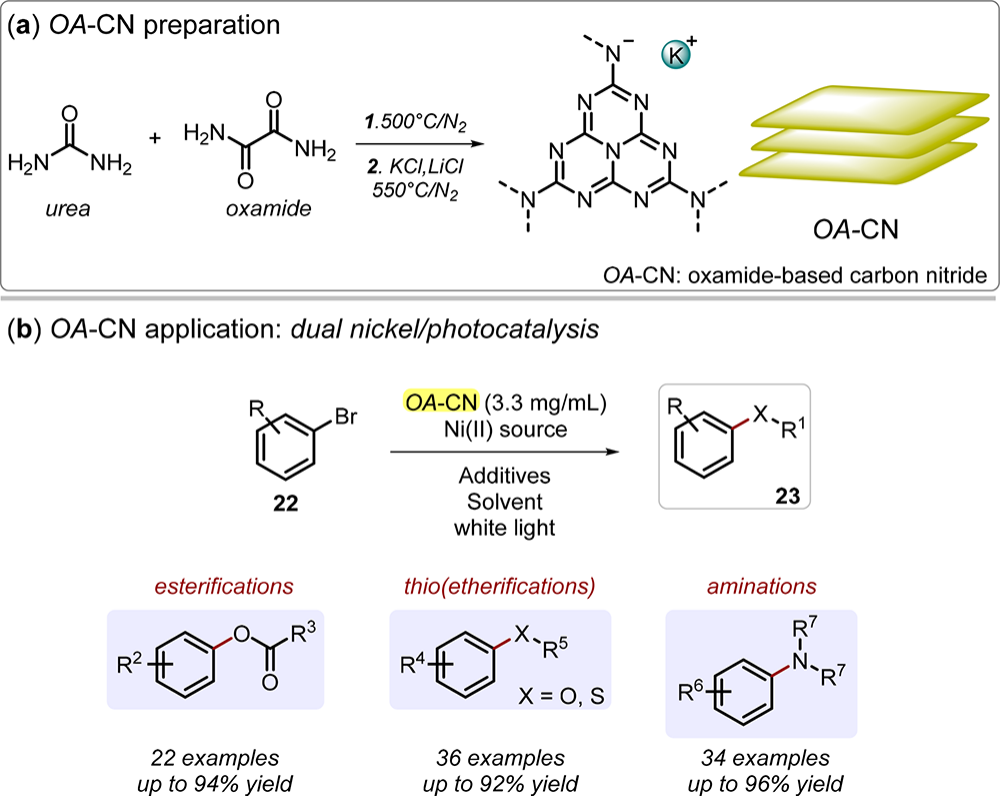
(a) OA-CN (oxamide-based carbon nitride) preparation from urea and oxamide through calcination. (b) OA-CN application in photocatalyzed esterification, (thio)etherification, and amination reactions by means of dual nickel/photocatalysis.

Another recent trend that we expect to flourish in the near future is coupling 2D metal-free materials with metal complexes. The concept is to make use of transition metal complexes for combining photocatalysis with conventional organic catalysis. More specifically, the photocatalyst is intended to serve as a single-electron-transfer (SET) shuttle to harness the metal complex with specific oxidation states and the coordination environment to perform the tasks required by the mechanism. Although not purely metal-free overall, this strategy still builds on the ability of metal-free materials to absorb light, to generate the separated excited charges, and to transfer the charges. Pioneering work by Durrant, Reisner, and co-workers coupled CN photocatalysts with Ni diphospine complexes to achieve the dual function of H_2_ solar generation and the simultaneous oxidation of benzyl alcohol.^88^ This work also inspires studies of more challenging purely organic reactions. Pieber and co-workers exploited this metal/OA-CN (oxamide-based carbon nitride) dual mechanism to enable a variety of organic reactions such as esterifications, (thio)etherifications, and aminations under white light irradiation (Figure 9),^89−92^ while Ghosh *et al.* used dual catalysis by Ni derivatives and mpg-CN to drive coupling of aryl halides with aliphatic/aromatic amines and sulfamides (Figure 3).^39^

Such examples are quite recent and exclusively include CN as the 2D photocatalyst. The key mechanistic aspects are not yet well understood and require further exploration, but it is likely that the specific steps will differentiate according to the type of reaction and catalytic system.^93^ Nevertheless, these examples highlight the immense potential for organic synthesis, possibly encompassing a wide range of reactivity. Future developments will depend on the study and definition of the main features of the catalytic cycle for each class of investigated reactions, such as, for example, the dynamics of the SET steps (whether it is a direct transfer, a second coordination sphere transfer, or a solvent-mediated transfer) and the nature of the metal active site.

To sum up, several examples of non-metal-based 2D materials beyond graphene have all the qualifications to satisfy the strict requirements of photocatalysis. Thus far, a great effort has focused on their use as components in hybrid catalytic systems, with widespread use in energy-related photocatalysis. However, photocatalytic organic synthesis is experiencing a great deal of attention, and we expect that it will take a central role for future applications of this class of materials. Future avenues of development of 2D metal-free catalysts will converge toward (i) the ability to control and to modify the structure by synthetic schemes; (ii) the appropriate advanced characterization tools and methods required to reveal the substructure/functionality relationships in photocatalysis; (iii) nonreliance on high metal loadings (*i.e*., exploring possible introduction of low fractions of single metal atoms); and (iv) the possibility of interfacing different 2D nonmetal phases with suitable contacts and interactions, both to extend the possible applicability to more demanding classes of reactions and to improve photocatalytic activity. More complex catalytic systems, such as the inclusion of metal species for performing traditional organic steps also represent an attractive opportunity, driving organic transformations of higher complexity.
